# Binding characteristics of the ovine membrane progesterone receptor alpha and expression of the receptor during the estrous cycle

**DOI:** 10.1186/1477-7827-7-42

**Published:** 2009-05-11

**Authors:** Ryan L Ashley, J Alejandro Arreguin-Arevalo, Terry M Nett

**Affiliations:** 1 Department of Biomedical Sciences, Animal Reproduction and Biotechnology Laboratory, Colorado State University, Colorado, USA

## Abstract

**Background:**

Classically, progesterone has been thought to act only through the well-known genomic pathway involving hormone binding to nuclear receptors and subsequent modulation of gene expression. However, there is increasing evidence for rapid, non-genomic effects of progesterone in a variety of mammalian tissues and it is possible that a membrane PR (mPR) is causing these events. We recently isolated and characterized an ovine mPR referred to as mPR-alpha, distinct from the nuclear PR. Based on predicted structural analysis, the ovine mPR-alpha possesses seven transmembrane domains typical of G protein-coupled receptors. Despite the homology to other reported mPRs, information pertaining to the steroid binding characteristics of the ovine mPR-alpha was lacking. Additionally, the ovine mPR-alpha transcript has been identified in the hypothalamus, pituitary, uterus, ovary and corpus luteum, yet changes in expression of the ovine mPR-alpha in these tissues were not known. Consequently, the purpose of this work was to determine the steroid binding characteristics of the ovine mPR-alpha and to investigate possible changes in expression of the ovine mPR-alpha in reproductive tissues throughout the estrous cycle.

**Methods:**

Binding studies were performed using crude membrane fractions from CHO cells expressing the mPR-alpha. Using quantitative Real-time PCR we determined the expression pattern of mRNA for the ovine mPR-alpha during the ovine estrous cycle in tissues known to express the mPR-alpha. Jugular blood samples were also collected and analyzed for serum concentrations of P4 to ensure ewes were at the appropriate stage of their cycle.

**Results:**

Only progesterone, 20alpha-hydroxyprogesterone and 17alpha-hydroxyprogesterone were able to displace binding of 3H-P4 (P < 0.001) to membrane fractions from CHO cells expressing ovine mPR-alpha. The average B-max and Kd values for three separate experiments were 624 +/- 119 fmol/micro gram protein and 122 +/- 50 nM, respectively. Significant changes in expression of mRNA for the mPR-alpha during the estrous cycle were noted in the corpus luteum and uterus.

**Conclusion:**

The mPR-alpha specifically binds progestins and its expression was correlated to progesterone secretion during the ovine estrous cycle. Results from the present studies suggest that mPR-alpha may have an important physiological role during the ovine estrous cycle.

## Background

Progesterone (P4) is a steroid hormone produced primarily by the ovary with the amount of P4 secreted depending on the level of gonadotropin stimulation and the physiological status of the ovary. Granulosa cells, theca/stromal cells and luteal cells all secrete P4 albeit at different levels [1-3]. Progesterone has many biological effects in a variety of tissues, and as such considerable research has focused on the mechanisms through which P4 mediates its actions. Many physiological effects of P4 are mediated through gene regulation by the nuclear P4 receptors (nPR) that function as ligand-dependent transcription factors. More recently, P4 also has been shown to evoke rapid stimulatory effects through a variety of signal transduction molecules and pathways (reviewed in [4]) and it is thought that these non-genomic effects are the result of P4 binding to a membrane PR (mPR). A unique mPR with seven hydrophobic amino acid domains indicative of a prototypical G protein-coupled receptor (GPCR) was recently cloned from the sheep [5] and shares significant homology to other reported mPRs [6-9]. Despite the homology of the ovine mPRα with mPRα from other species, information pertaining to the steroid binding characteristics of the ovine mPRα is lacking. An essential criterion to verify the putative ovine mPRα as a true receptor is to identify the ligand(s) that binds to the ovine mPRα and to determine the binding kinetics of this putative receptor. The first mPR isolated was from seatrout and these researchers utilized soluble recombinant protein produced in BL21 *E. coli *cells to determine the steroid binding characteristics [9]. Saturable binding of P4 was obtained with the recombinant protein and competition studies revealed binding was highly specific for progestins [9]. Similar results were obtained with recombinant human mPRα, mouse mPRβ and human mPRγ produced in an *E. coli *expression system [8].

Use of recombinant protein for binding studies provides researchers with a fairly straightforward method to evaluate interaction between ligand and receptor. However, limitations exist with this system as the protein synthesized may lack the normal tertiary structure and may lack post-translational modifications that would be present in situ. As such, the binding characteristics of the reported mPRs may be different in vivo than those obtained using a soluble form of the receptor. A paper investigating the regulation of membrane progestin receptors in the rat corpus luteum (CL) provides a better understanding of the binding characteristics of the mammalian mPRs [6]. Binding of [^3^H]-progesterone was evaluated in subcellular fractions of corpora lutea obtained from rats on d 14 of pregnancy. It was found that various progestins competed for binding of the radiolabeled P4 [6] and since these binding studies were performed with luteal membranes, the results may provide a better representation of the binding kinetics in vivo. These researchers also reported the presence of five genes expressed in the rat CL previously postulated to encode for putative membrane receptors for P4 [6]. Thus, it is difficult to verify which, or if all, of these proteins actually bind P4.

Coupled with determining the binding kinetics of the ovine mPRα, deciphering possible changes in expression of this receptor in vivo is also critical to help define the physiological role of the ovine mPRα. Changes in the level of expression of the mammalian mPRα have been demonstrated in the rat CL during pregnancy [6], human endometrium [10], human cervix and myometrium [11], and in human myometrial cells following exposure to P4 or estradiol [7]. Currently, the only tissues known to express the ovine mPRα are the hypothalamus, pituitary, uterus, ovary and CL [5]. Whether the level of expression of the ovine mPRα in these tissues changes throughout the estrous cycle is not known. Given that the ovine mPRα transcript is found predominately in reproductive tissues, elucidating possible changes in levels of mPRα expression throughout the ovine estrous cycle is paramount.

Experiments were conducted to test the hypothesis that P4 is the true ligand for the ovine mPRα and that levels of transcript for the ovine mPRα in reproductive tissues fluctuate depending on the stage of the estrous cycle. Consequently, in this study we determined the steroid binding characteristics of the ovine mPRα, by performing binding studies using solubilized crude membrane fractions from Chinese hamster ovarian (CHO) cells expressing the sheep mPRα. By using a mammalian cell line, the post-translational modifications of the receptor should be unaltered, yet the tertiary structure may be affected by solubilization. Changes in levels of expression of the ovine mPRα were also examined in the hypothalamus, pituitary, uterus, non-luteal components of the ovary, and CL throughout the estrous cycle.

## Methods

### Saturation binding experiments and competition curves

A stable cell line which expresses the ovine mPRα was generated for the binding studies involving ligand saturation and competition curves. CHO cells were transfected with a vector (pcDNA3.1+) encoding ovine mPRα using the Polyfect procedure (QIAGEN, Valencia, California, USA) according to manufacturer's instructions. Twenty four hours after transfection, the medium was changed, and G418 (Mediatech, Inc., Herndon, Virginia, USA) was added to a final concentration of 400 μg/mL. After 2 weeks of selection in G418, the cells were trypsinized and serially diluted in complete medium plus G418, plated into wells of a 96-well tissue culture plate, and incubated until single colonies became visible. Each well that contained a single colony was grown to confluence at which time colonies were trypsinized and transferred to a single well of a 24-well tissue culture plate. Once these colonies reached confluence, individual colonies were trypsinized and each colony was cultured in a single well of a 6-well tissue culture plate. The same procedure was performed again, but colonies were seeded onto a 90 mm tissue culture dish. Expression of the ovine mPRα was determined in each colony by RT-PCR for the ovine mPRα using procedures described by Ashley and colleagues [5]. Membrane fractions from the stable CHO cell line expressing ovine mPRα were incubated with increasing concentrations of ^3^H-P4 for the saturation binding experiments. The binding studies were performed as described below in the "Receptor Binding Assays" section, except that the incubation time was increased to 4 h at 4°C based on the time course experiments. Data from the saturation experiments were analyzed by nonlinear regression analysis using GraphPad Prism (Version 4a, from GraphPad Software, Inc.). Binding of ^3^H-P4 was also measured in the presence of increasing concentrations of P4, 20α-hydroxyprogesterone or 17α-hydroxyprogesterone to determine the half-maximal inhibitory concentration (IC_50_) of these progestins. The IC_50 _of the progestins was obtained by nonlinear fitting of inhibition curves using GraphPad Prism (Version 4a, from GraphPad Software, Inc.).

### Receptor Binding Assays

A crude membrane fraction was prepared from CHO cells and CHO cells transfected with cDNA for ovine mPRα following procedures as described [12] with slight modifications. CHO cells transfected with a vector (pcDNA3.1+) encoding ovine mPRα using the Polyfect procedure (QIAGEN, Valencia, California, USA) were cultured in DMEM, supplemented with 10% FBS, non-essential amino acids (8.9 mg/L L-Alanine, 15 mg/L L-Asparagine, 13.3 mg/L L-Aspartic acid, 14.7 mg/L L-Glutamic acid, 7.5 mg/L Glycine, 11.5 mg/L L-Proline and 10.5 mg/L L-Serine) and penicillin-streptomycin (100 I.U. penicillin and 100 μg/mL streptomycin). At 48 h post-transfection, transfected and non-transfected CHO cells were washed with cold PBS and removed from tissue culture plates using a cell scraper and cold PBS, and concentrated by centrifugation. Cells were washed again with cold PBS, counted using a hemacytometer and lysed in homogenization buffer (100 mM KCl, 5 mM MgCl_2_, 50 mM Tris-HCl, 1 mM EGTA, plus protease inhibitors; pH 7.2) with a QIAshredder (QIAGEN, Valencia, CA) per manufacture's instructions. Supernatants were collected and spun at 100,000 × g at 4°C for 1 h. Supernatants were removed and pellets containing the membrane fractions were resuspended in cold sample buffer (10 mM Tris-HCl, 250 mM sucrose, 1 mM EGTA, plus protease inhibitors; pH 7.2). An aliquot of the membrane fractions from transfected and nontransfected cells was kept separate and protein concentration was determined using the Coomassie Plus Protein Assay Kit (Pierce; Rockford, Illinois, USA). Duplicate aliquots of membrane fractions from CHO cells transfected with mPRα and from nontransfected CHO cells were incubated at 4°C for 1 h in 0.3 mL TEDG buffer (10 mM Tris-HCl, 1.5 mM EDTA, 1 mM Dithiothieitol, 10% Glycerol; pH 7.6) containing 4 nM ^3^H-progesterone (^3^H-P4) and 250 μM digitonin. Bound and free ligands were separated by the addition of 0.8 mL ice cold dextran-coated charcoal (0.3 g defined charcoal and 0.03 g Dextran [Sigma, St Louis, MO] in 100 mL of TEDG buffer) and incubation on ice for 10 min. After centrifugation at 1100 × g for 15 min at 4°C, 0.9 mL supernatants were carefully removed, mixed with 5 mL of ScintiSafe scintillation cocktail (Fisher Chemical, Fair Lawn, New Jersey, USA) and radioactivity was quantified in a Beckman scintillation spectrometer. Non-specific binding was measured in duplicate in the presence of a 1000-fold excess of nonradioactive P4. Other binding experiments were performed with increasing concentration of protein and increasing incubation time. Additional controls included tubes without cell membrane fractions but with ^3^H-P4 and digitonin. To test the specificity of binding for the ovine mPRα, binding of ^3^H-P4 was measured in the presence of 1000-fold excess of several other nonradioactive steroids. Statistical analysis of the results from the competition binding studies were performed using the Newman-Keuls Multiple Comparison Test in Prism (Version 4a, from GraphPad Software, Inc.) and significance was taken as a value of *P *< 0.05.

### Relative expression of mRNA for ovine mPRα throughout the estrous cycle

All procedures involving animals were approved by the Colorado State University Animal Care and Use Committee. Ewes were exposed to a vasectomized ram to determine the onset of estrus. On day 13–14 of the cycle, ewes were administered dinoprost tromethamine (Lutalyse) (7.5 mg I.M.) two times, 4 h apart for estrus synchronization. Before the first dose of Lutalyse, jugular blood samples were taken from each ewe for analysis of serum concentrations of P4. Jugular blood samples were also taken daily from each ewe until time of euthanasia. Blood was allowed to clot for 1 h at room temperature and serum was collected by centrifugation. Serum was stored at -20°C until analyzed for concentrations of P4. Following Lutalyse treatment, ewes were observed continually in the presence of a vasectomized ram until estrus was detected, at which time ewes were subsequently assigned to various groups depending on when estrus was first observed. Groups consisted of 3 h and 24 h post onset of estrus and days 4, 10, and 15 of the estrous cycle. At the appropriate time of the cycle, tissues (hypothalamus, pituitary, whole uterine sections, corpora lutea, and ovaries minus corpora lutea) were excised from sheep following euthanasia with sodium pentobarbitol and exsanguination, and snap frozen in liquid nitrogen for subsequent isolation of RNA. Total RNA was extracted using the RNeasy Midi Kit (QIAGEN, Valencia, California, USA) according to manufacturer's instructions. To ensure RNA samples were not contaminated with genomic DNA, each sample was subjected to RNase-Free DNase treatment (QIAGEN, Valencia, CA). Concentration of RNA was determined by spectrophotometry, and integrity of RNA verified by gel electrophoresis in 1% agarose in the presence of ethidium bromide followed by visualization under UV light. A set concentration of RNA (1 μg) was reverse-transcribed into cDNA using the iScript cDNA Synthesis Kit employing the reverse transcriptase RNAse H+ (Bio-Rad Laboratories, Hercules, CA) per manufacturer's instructions. The RT products were diluted to a final volume of 100 μL. Quantitative PCR was performed using a Bio-Rad iCycler iQ Real-Time PCR Detection System (Bio-Rad Laboratories, Hercules, CA). Real-time quantitative PCR was performed in single wells of a 96-well plate (Bio-Rad Laboratories, Hercules, CA) using components of the iQ SYBR Green supermix (2×), 0.5 μM forward and reverse specific primers, and 10 μL of the RT product. As a negative control for all the reactions, preparations lacking RNA or reverse transcriptase were used in place of the cDNA. The following primers were used for PCR amplification: Ovine mPRα, TTGTGGGCACCGTGGACTTC and GCTAAGGCACTGAGGGAGAGG; Ovine β-actin TCTGGCACCACACCTTCTAC and GGTCATCTTCTCACGGTTGG. The following PCR thermocycling program was used: 95°C for 3 min, followed by 40 cycles consisting of 95°C for 10 sec and 55°C for 45 sec, followed by one cycle each of 95°C for 1 min, and 55°C for 1 min and a melt curve. Fluorescence was measured after each cycle and displayed graphically (iCycler iQ Real-Time Detection System Software, version 3.1; Bio-Rad). The software determined the cycle threshold (Ct) values for each sample. Data were normalized by subtracting the Ct value of actin from the Ct value of mPRα for each ewe on each day tested. Sample RNAs were assayed from three to five independent biological replicates. Statistical analysis was performed on the normalized data using the Newman-Keuls Multiple Comparison Test in Prism (Version 4a, from GraphPad Software, Inc.) and significance was taken as a value of *P *< 0.05. When a significant difference (*P *< 0.05) was noted, the RNA levels were reported as a fold change in the text, using the "Delta-delta method" for comparing relative expression results in real-time PCR [13]. Data are shown as the mean values (actin Ct/mPRα Ct) ± S.E.

### Radioimmunoassay for serum progesterone

The concentration of P4 in serum from the ewes was quantified by a double antibody radioimmunoassay [14]. Triplicate standard curves were included in each assay and samples were analyzed in duplicate at 200 μL sample/tube.

## Results

**Figure 1:**
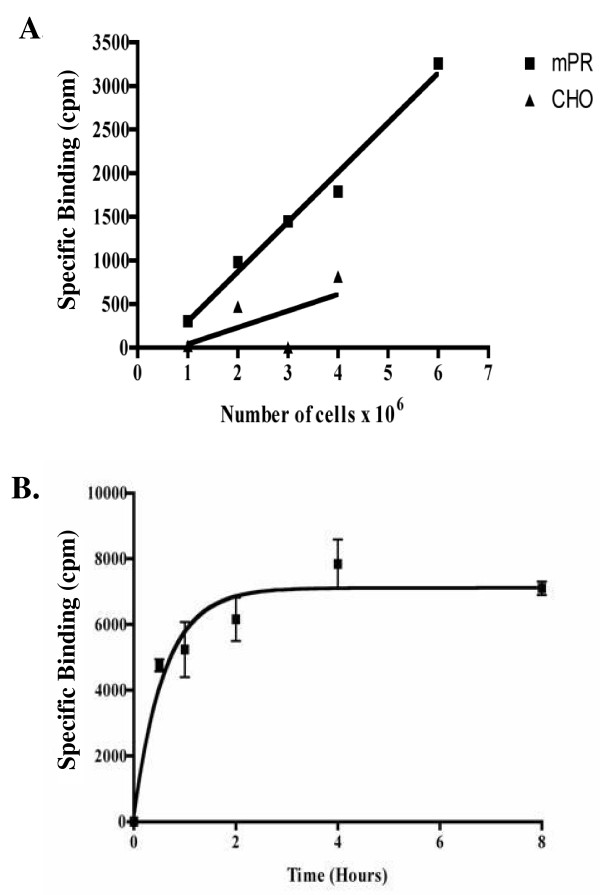
**A) Binding of ^3^H-progesterone (cpm) was measured in increasing amounts of crude membranes from CHO cells and CHO cells expressing mPRα**. Displacement of ^3^H-progesterone was measured in the presence of 1000-fold excess nonradioactive progesterone. Values are a representation of three individual experiments. B) Binding of ^3^H-Progesterone in crude membrane fractions from CHO cells expressing the ovine mPRα at various times of incubation. Displacement of ^3^H-progesterone was measured in the presence of 1000-fold excess nonradioactive progesterone. Values are means ± S.E. (n = 4).

**Figure 2:**
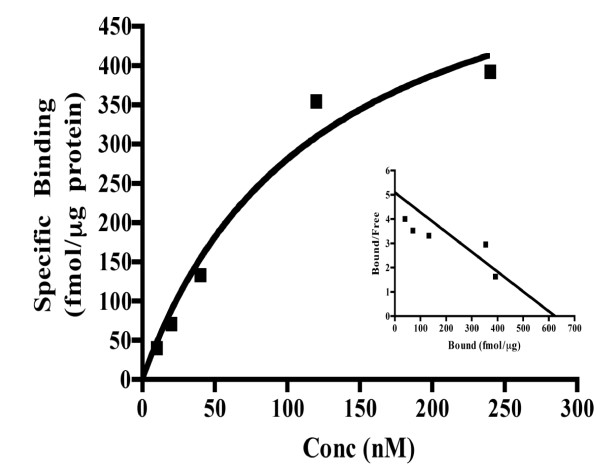
**Representative saturation curve in the presence of increasing concentrations of ^3^H-progesterone**. The B_max _and K_d _values of ^3^H-progesterone binding were 624 fmol/μg protein and 122 nM, respectively. The *inset *shows Scatchard analysis of the same data.

As shown in Figure 1A, binding of ^3^H-P4 increased with increasing amounts of membrane fractions from mPRα-CHO cells. In contrast little binding was observed in the membrane fractions from CHO cells. Likewise, binding of ^3^H-P4 increased with incubation time reaching a plateau at approximately 4 h at 4°C (Figure 1B). A representative saturation curve of ^3^H-P4 binding to membrane fractions from mPRα-CHO cells is shown (Figure 2). The average B_max _and K_d _values of three separate experiments were 624 ± 119 fmol/μg protein and 122 ± 50 nM, respectively.

**Figure 3:**
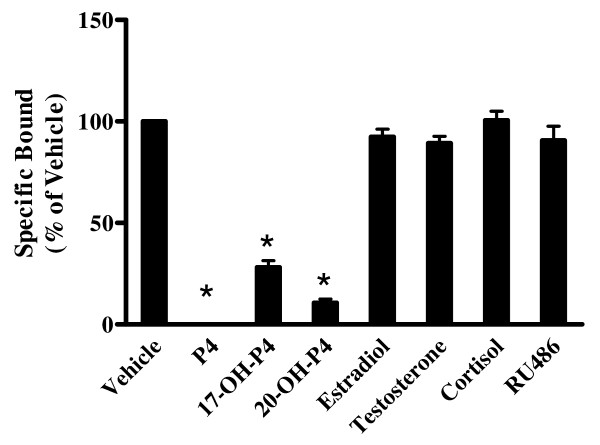
**Displacement of ^3^H-progesterone from crude membranes of CHO cells expressing mPRα in the presence of vehicle or 1000-fold excess of nonradioactive steroids**. Steroids included progesterone (P4), 17α-hydroxyprogesterone (17-OH-P4), 20α-hydroxyprogesterone (20-OH-P4), estradiol, testosterone, cortisol, or RU486. Values are means ± S.E. (n = 3). *P < 0.001, compared to vehicle control.

A variety of steroids were tested (1000-fold excess) for their abilities to displace binding of ^3^H-P4 to crude membrane fractions of mPRα transfected CHO cells. Only excess P4, 20α-hydroxyprogesterone and 17α-hydroxyprogesterone were able to significantly (*P *< 0.001) displace binding of radiolabeled P4. Estradiol, testosterone, cortisol and the P4 antagonist RU486 failed to inhibit binding of ^3^H-P4 to crude membranes from CHO cells expressing mPRα (Figure 3).

**Figure 4:**
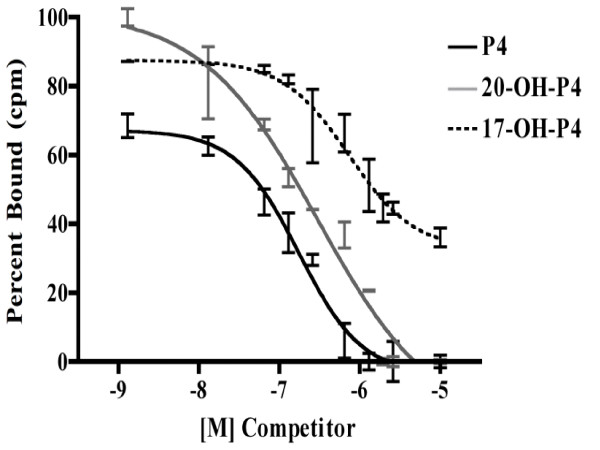
**Specificity of progestin binding sites for ovine mPRα**. Binding of ^3^H-progesterone was measured in the absence or in the presence of increasing concentrations of nonradioactive progestins. Progestins evaluated included progesterone (P4), 20α-hydroxyprogesterone (20-OH-P4) and 17α-hydroxyprogesterone (17-OH-P4). Each point represents the average ± S.D. of progestin binding expressed as a percentage of maximum ^3^H-progesterone binding.

Based on the competition binding studies, additional experiments were performed to more clearly evaluate the abilities of P4, 20α-hydroxyprogesterone and 17α-hydroxyprogesterone to displace binding of radiolabeled P4 to crude membranes from mPRα-CHO cells (Figure 4). Low levels of P4 (IC_50 _= 174 nM) competed for binding of the tracer in a dose-dependent manner (Figure 4), whereas higher concentrations of 20α-hydroxyprogesterone (IC_50 _= 298 nM) or 17α-hydroxyprogesterone (IC_50 _= 735 nM) were required to reduce binding by 50% (Figure 4).

**Figure 5:**
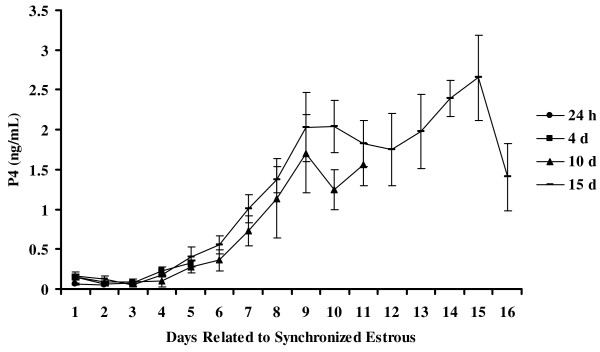
**Serum concentrations (ng/mL) of P4 in synchronized ewes**. Five ewes were assigned to each time period and synchronized with Lutalyse (7.5 mg I.M.) injections two times, 4 h apart. Time periods included 3 h and 24 h post estrus and days 4, 10, and 15 of the estrous cycle. For clarity, serum concentrations of P4 are only shown from onset of estrus (d 1) to d 15. Data are means ± S.E. (n = 5 ewes/time period).

**Figure 6:**
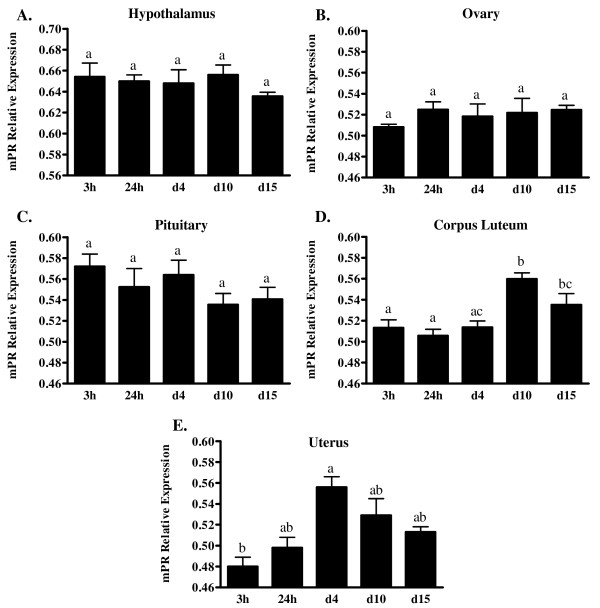
**Comparison of mean relative expression of mRNA encoding mPRα versus mRNA encoding actin in ovine A) hypothalamus, B) non-luteal components of the ovary, C) pituitary, D) corpus luteum, or E) uterus throughout the estrous cycle (3 h and 24 h post estrus and days 4, 10, 15)**. Values on the y-axis (actin Ct/mPRα Ct) represent means ± SE of a minimum of three sheep. Significant differences (*P *< 0.05) between groups are indicated by different letters above the bars.

To determine if there were possible changes in expression of mPRα throughout the ovine estrous cycle, ewes were euthanized and tissues were collected at 3 h and 24 h post onset of estrus and then at days 4, 10, and 15 of the estrous cycle. Jugular blood samples were also collected and analyzed for serum concentrations of P4 to ensure ewes were at the appropriate stage of their cycle. As shown in Figure 5 all of the ewes appeared to be synchronized and displayed low serum concentrations of P4 at the beginning of their cycle; levels then increased to maximal concentrations on d 8 and then began to decline at the end of the cycle. No apparent differences were observed for mean relative expression of mRNA for mPRα in hypothalamus (Figure 6A), the non-luteal component of the ovary (Figure 6B), or pituitary (Figure 6C) during the estrous cycle. In contrast, in the CL, the highest expression of mRNA for mPRα was observed on d 10 and was 3.9, 4.5 and 2.5 fold greater (*P *< 0.01) than expression on d 4 or at 3 h and 24 h post estrus, respectively (Figure 6D). Additionally, the expression of mPRα in the CL was 2.2 and 2.6 fold greater (*P *< 0.05) on d 15 compared to 3 h and 24 h post estrus, respectively (Figure 6D). The highest expression of mRNA for mPRα in the uterus was on d 4 when it was 3.7 fold higher (*P *< 0.01) than mRNA at 3 h post onset of estrus (Figure 6E).

## Discussion

Our studies demonstrate binding of ^3^H-P4 in membrane fractions from cells expressing the ovine mPRα. Currently, limited binding data for the mammalian mPRα homologs exists. Only P4, 20α-hydroxyprogesterone and 17α-hydroxyprogesterone significantly (*P *< 0.001) displaced binding of ^3^H-P4 to membrane fractions from CHO cells expressing ovine mPRα. Similar to the ovine mPRα, the seatrout mPRα also exhibits specific binding for P4, 20β-hydroxyprogesterone and 17α-hydroxyprogesterone [9]. Additionally, MDA-231 cells expressing the human mPRα also display specific binding for P4 [15]. Other steroids tested in the current study did not compete for ^3^H-P4 binding further supporting progestins as the true ligand for the ovine mPRα. It is important to note the membrane fractions used in the present study are comprised of the plasma membrane as well as membranes from other cellular organelles. Specific binding sites for P4 have been detected in microsomal rich fractions in a variety of mammalian species. Bramley and Menzies [16] reported P4 binding sites in ovine CL that are unlike classical nuclear PRs in that they are enriched in intracellular membrane fractions and not found in the cytosolic or nuclear fractions. Also, in those studies, RU486, the nuclear PR antagonist did not block P4 binding to intracellular membranes suggestive of a PR unlike the nPR [16]. Likewise, in the present study RU486 failed to compete for ^3^H-P4 binding in membrane fractions from CHO cells expressing ovine mPRα. Similar results have been reported for microsomal fractions of corpora lutea from cows [17], pigs [18], humans [19] and rat [20]. These data provide further support for a PR functionally distinct from the classic nuclear PR with predominant localization in an intracellular membrane such as the endoplasmic reticulum. We previously demonstrated that the ovine mPRα is predominantly localized in the endoplasmic reticulum and upon ligand activation causes a release of Ca^2+ ^from the endoplasmic reticulum [5]. While we cannot for certain rule out that cellular localization of the receptor may be altered in CHO cells, we have performed a plethora of studies using mPRα fusion proteins in primary pituitary and luteal cells and have observed the same localization of mPRα in these cells as noted in CHO cells. This unique localization to the endoplasmic reticulum has since been corroborated in cells expressing the human mPRα [21].

The apparent dissociation constant of 122 nM from our saturation experiments is well within the range of values reported for other membrane P4-binding proteins, including those found in bovine CL [197 nM] [17], rat liver [170 nM] [22] porcine liver [11–286 nM] [23] and the rat brain [160 nM] [24]. Additionally, in a paper investigating the various receptors for P4 in rat CL, which contains the mPRα homolog, an apparent dissociation constant of 162 nM was observed [6]. Although the ovine mPRα has a relatively low affinity, given its expression in the CL, it is plausible that luteal membrane P4-binding sites are completely saturated because of the high intraluteal concentration of P4. Additionally, to date very little data are available pertaining to the binding characteristics of the mammalian mPR homologs. Information presented in this paper coupled with the binding studies in membranes from rat CL [6] and MDA-MB-231 breast carcinoma cells transfected with cDNA encoding the human mPRα [15] provide some of the first data describing the binding characteristics of the mammalian mPRs.

In terms of binding kinetics, the ovine mPRα shares greater similarities with the rat mPR than the human or non-mammalian homolog. The K_d _reported in membrane fractions from rat CL is 162 nM which is similar to the 122 nM K_d _for the ovine mPRα. It is noteworthy to mention that the rat CL expresses 5 genes which encode for putative membrane P4 receptors [6]. Thus, direct comparison of our data with that from the rat is difficult because it is not known how many of the proteins in the rat CL are expressed and may bind P4. The current ovine mPRα and the recent human mPRα binding data [15] are the first studies to demonstrate and characterize progestin binding in cells expressing the mPRα homolog alone. The human mPRα displays binding kinetics that differs from the ovine receptor as Thomas and colleagues demonstrated that the human mPRα has a Kd of 4.2 nM. The reasons for the differences in the Kd are not known but may be due to structural differences among species or to experimental protocols. Nevertheless, the relatively low affinity for the ovine mPRα plus the data demonstrating the lack of RU486 binding provide further evidence that the mammalian mPRs appear to function in a manner distinct from the nPRs.

As the ovine mPRα transcript is predominately found in reproductive tissues [5], we decided to investigate possible changes of mPRα expression throughout the ovine estrous cycle in these tissues. Using real-time quantitative RT-PCR, no significant changes were observed across the estrous cycle in amounts of mRNA for mPRα in the hypothalamus, non-luteal components of the ovary, or pituitary. Despite no significant changes across time in the hypothalamus, relative expression of mPRα did appear to be higher in the hypothalamus compared to other tissues tested. In the ovine CL, mRNA for the mPRα increased from estrus throughout the cycle and was significantly (*P *< 0.01) higher on d 10 compared to 3 h and 24 h post onset of estrus or d 4 of the cycle (Figure 6D). The expression of mPRα in the CL remained elevated on d 15 and was significantly (*P *< 0.05) higher compared to 3 h and 24 h post onset of estrus (Figure 6D). It is important to note, that CL samples taken from sheep at 3 h and 24 h post onset of estrus in actuality represent the corpus albicans. As noted, relative expression of the mPRα was significantly (*P *< 0.05) lower in the corpus albicans samples compared to d 10 and d 15 samples, providing support for a functional role for mPRα in the CL. Comparing serum concentrations of P4 (Figure 5) to the expression of mPRα in the CL (Figure 6D), it is evident that expression of mPRα closely mimics serum concentrations of P4 suggesting that the ovine mPRα may be regulated by P4 and that the mPRα may play a central role in the regulation of CL function. The only other data available regarding mPRα expression in the CL is from the rat [6]. These researchers however, measured changes in mRNA throughout pregnancy in the rat CL and observed that mPRα expression increased with advancing gestation and then dramatically decreased prior to parturition. Thus, similar to our observations expression of mPRα in the rat CL also mimicked serum concentrations of P4 [6] further suggesting that mPRα plays a key role in CL function.

As with changes in levels of expression of mPRα in the CL, very little data exist pertaining to changes in expression of the mammalian mPRα in other tissues. Karteris et al. [7] demonstrated that P4 and estradiol significantly induced expression of mRNA for human mPRα in human myometrial cells. Additionally, expression of mRNA for the human mPRα was reported throughout the myometrium with peak levels observed in spontaneously laboring women [11]. However, this observation is in direct contrast to Fernandes et al. [10] who described a 50% reduction in mRNA for mPRα in lower segment myometrium from spontaneously laboring women. In the current study, uterine mRNA on d 4 of the estrous cycle contained the highest levels of expression for the ovine mPRα. The expression of the mPRα was significantly (*P *< 0.01) higher on d 4 of the estrous cycle compared to 3 h post onset of estrus with an approximate 4 fold increase in mRNA (Figure 6E). Although not significantly different, the expression of mPRα appears to decrease sequentially from d 4 to days 10 and 15 (Figure 6E). Though estradiol was not assayed in our serum samples, the difference in expression of mPRα noted between 3 h post onset of estrus and d 4 may be due to changes in the ratio of circulating P4 and estradiol. During the estrous cycle it is generally thought that early in the cycle concentrations of estradiol in blood are higher compared to P4 until the CL increases production and secretion of P4. As concentrations of P4 increase in circulation throughout the luteal phase of the cycle, estradiol levels decrease. As such, at 3 h post onset of estrus the ratio of estradiol to P4 in circulation should be higher than on d 4 of the cycle when estradiol is low and serum concentrations of P4 are increasing. Whether the differences in expression of the ovine mPRα throughout the estrous cycle are due to changes in the serum concentrations of P4 and estradiol is currently not known and future studies are designed to address this issue.

In addition to the alpha homolog of the mPR, two other mPRs termed beta and gamma also exist in mammals. The current study detailed expression and binding characteristics of mPRα as this was the first mPR we isolated from sheep [5]. However, we have recently demonstrated expression of mRNA for both mPRβ and mPRγ in the CL and endometrium of sheep (unpublished data) and future studies are aimed at investigating the functions of all mPRs in these tissues. Further, we have also isolated the progesterone membrane receptor component 1 (PGMRC1) from ovine CL (unpublished data) which was originally purified from porcine tissue [23,25]. The PGMRC1 is expressed in rat CL during pregnancy [6] and its potential role in mediating antiapoptotic actions of P4 in spontaneously immortalized granulosa cells has been investigated in detail by Peluso and co-workers [26]. The precise functions of PGMRC1 and the other mPRs in the sheep are currently not known, but studies are on-going to decipher the physiological functions of these receptors.

## Conclusion

In summary, the binding characteristics of the ovine mPRα and the first characterization of expression of the ovine mPRα throughout the estrous cycle in the hypothalamus, pituitary, uterus, non-luteal components of the ovary, and CL are presented. Past studies have reported binding of P4 within microsomal fractions from corpora lutea of a variety of mammalian species, [18,19,27-29], however, the protein interacting with P4 was not elucidated. The ovine mPRα is the first mammalian protein identified that both localizes to the endoplasmic reticulum [5] and binds P4. The studies presented herein describe the in vivo profile for changes in expression of mPRα in the hypothalamus, pituitary, non-luteal components of the ovary, uterus, and CL during the ovine estrous cycle. Characterizing fluctuations in expression may aid in helping to define the physiological functions of mPRα. While further studies are required to characterize the actions of mPRα, the expression of this atypical P4 receptor provides an exciting venue for research into mediation of non-genomic effects of P4. Recently, mPRs have been reported to activate G_i _[15]. If this is true for the ovine mPRα, it could be involved in mediating a rapid negative effect of P4 on GnRH/LH secretion via interaction with receptors in the hypothalamus and/or anterior pituitary gland. The existence of a unique form of P4 receptor that contains seven transmembrane domains and resides in the endoplasmic reticulum is exciting and fuels enthusiasm for further research into the mode of action for this receptor. This novel method of signaling at the endoplasmic reticulum and the existence of multiple ligands for the ovine mPRα adds to the intricacy of signaling in cells and provides a mechanistically unique method for initiating actions of progestins that may alter classical dogma regarding the mechanisms by which steroid hormones act.

## Competing interests

The authors declare that they have no competing interests.

## Authors' contributions

All authors contributed to the overall design of the study. JAAA performed the assay for serum concentrations of progesterone. RLA performed all the receptor binding and qRT-PCR studies and drafted the manuscript. All authors read and approved the manuscript.
